# Supplementary material for: Integrative Investigation of Root-Related mRNAs, lncRNAs and circRNAs of “Muscat Hamburg” (Vitis vinifera L.) Grapevine in Response to Root Restriction through Transcriptomic Analyses
Source: Genes (Basel). 2022 Aug 27;13(9):1547. doi: 10.3390/genes13091547 (PMC9498474; doi:10.3390/genes13091547)
Supplement: Supplementary file 1 [file genes-13-01547-s001.zip › genes-1839530-supplementary.pdf]

Supplementary Materials

Table S1. Divergent primers and convergent primers used for detection the expression profiling of circRNA_2377

Gene name	Divergent primers	Convergent primers
circRNA_2377	F: CGACTGATGCTTGCTGAAAGA	F: TAGGATCTACTGAGTGGGTCG
	R: GGACATAGCGATCAGGCTCT	R: TAGCCTCTGGATATTGATGACTT

Table S2. Screening of candidate genes related to root development by high-throughput sequencing based on GO enrichment

**Gene name**	**GO_id**	**GO_term**
VIT_06s0061g00310	GO:0005886,GO:0009705,GO:0010167,GO:0015112,GO:0015706,GO:0016020,GO:0016021,GO:0042128,GO:0048527,GO:0071249	Plasma membrane, plant-type vacuole membrane, response to nitrate, nitrate transmembrane transporter activity, nitrate transport, membrane, integral component of membrane, nitrate assimilation, **lateral root development**, cellular response to nitrate
TOCNS_00012993	GO:0005886,GO:0009630,GO:0016567,GO:0046872,GO:0048364,GO:0061630	plasma membrane, gravitropism, protein ubiquitination, metal ion binding, **root development**, ubiquitin protein ligase activity
TOCNS_00037367	GO:0003774,GO:0003779,GO:0005516,GO:0005524,GO:0007015,GO:0009506,GO:0010090,GO:0010091,GO:0016459,GO:0030048,GO:0030133,GO:0035619,GO:0048467,GO:0048767,GO:0048768,GO:0051645,GO:0051646,GO:0060151,GO:0090436	motor activity, actin binding, calmodulin binding, ATP binding, actin filament organization, plasmodesma, trichome morphogenesis, trichome branching, myosin complex, actin filament-based movement, transport vesicle, **root hair tip**, gynoecium development, **root hair elongation**, **root hair cell tip growth**, Golgi localization, mitochondrion localization, peroxisome localization, leaf pavement cell development
circRNA_2377	GO:0004180,GO:0004181,GO:0005789,GO:0006508,GO:0009640,GO:0009793,GO:0009908,GO:0010080,GO:0010081,GO:0010082,GO:0010305,GO:0016021,GO:0046872,GO:0048364,GO:0048507	carboxypeptidase activity, metallocarboxypeptidase activity, endoplasmic reticulum membrane, proteolysis, photomorphogenesis, embryo development ending in seed dormancy, flower development, regulation of floral meristem growth, regulation of inflorescence meristem growth, **regulation of root meristem growth**, leaf vascular tissue pattern formation, integral component of membrane, metal ion binding, **root development**, meristem development

Table S3. Sequences of primers used for real-time PCR

Gene name	Forward primer (5’- 3’)	Reverse primer (5’- 3’)
VIT_06s0061g00310	ATGCTCTTCTCCGTTGGAGC	TCAGTCCGGAGCCAAAGTTC
TOCNS_00012993	CAAACAGGGCGTTTTGGAGAAG	TGCTCTGATTCATACCCGTCTT
TOCNS_00037367	GATAAGCAGCAGGTGGCTGA	GTGGCAAGTACCTAAGGCCA
circRNA_2377	CGACTGATGCTTGCTGAAAGA	GGACATAGCGATCAGGCTCT
